# Special Section: Multidisciplinary Perspectives on Challenges in Managing Smart Products and Services

**DOI:** 10.1007/s41464-020-00104-z

**Published:** 2020-11-05

**Authors:** Nicola Bilstein, Christian Stummer

**Affiliations:** Faculty of Business Administration and Economics, Bielefeld University, Universitätsstr. 25, 33615 Bielefeld, Germany

“The future will be characterized by smart devices delivering increasingly insightful digital services everywhere” predicts David Cearley, Gartner Distinguished Vice President Analyst (Panetta 2018). Today’s advances in information and communication technologies already allow transforming traditional consumer products into smart products and offering novel smart services (Dawid et al. 2017; Wünderlich et al. 2015). Such smart products can be understood as cyber-physical devices consisting of both tangible (i.e., hardware) and intangible (i.e., software) components that may render smart services and may operate within a larger ecosystem (Raff et al. 2020).

The emergence of smart products and services leads to various managerial challenges. Consequently, novel research questions, for example, with respect to additional or possibly different drivers and barriers of product adoption emerge (Mani and Chouk 2018; Schweitzer and van den Hende 2016; Souka et al. 2020). The effect of the anthropomorphism of a smart product on purchase intention may serve as an illustrative example. Further, new markets/services might be created (Porter and Heppelmann 2014) such as mobile location-based services that provide value-added services to consumers in inner cities with respect to their geographic context and individual preferences. Smart products and services may also demand for innovative, data-driven business models (Ng and Walkenshaw 2017): smart energy products, for example, generate large volumes of data for which the business value is still unclear. Finally, smart products and services may transform companies (Porter and Heppelmann 2015): implementing smart service innovation, for example, usually cannot be accomplished as an internal project but requires network management activities.

Given the diversity of challenges and the scarcity of research on the subject, we organized the First International Conference on Challenges in Managing Smart Products and Services (CHIMSPAS) at Bielefeld University, Germany, in August 2019. After the conference, we invited participants as well as other researchers from the field to submit their work to this Special Section and, thus, to contribute to a more comprehensive picture of the particularities related to the management of smart products and services. In response, we received insightful research from multidisciplinary perspectives on this topic addressing the multiple challenges faced by companies due to the transition from traditional products and services toward smart products and services. Some of the submitted papers, though very promising, were in a too early stage for publication, such that only four articles finally made it successfully through the rigorous review process.

The first article, “Mechanisms and consequences of anthropomorphizing autonomous products: The role of schema congruity and prior experience” by Moritz Jörling, Robert Böhm, and Stefanie Paluch, scrutinizes the meaning of anthropomorphization for the design of autonomous products (i.e., smart products supporting consumers in everyday tasks) and its implications for consumers’ perceptions and intentions. Building on schema congruity theory (Fiske and Linville 1980), the authors reveal in two online studies and one lab study that anthropomorphism is perceived more congruent with autonomous than manual products and therefore may increase the liking of autonomous products. Increased liking strengthens in turn consumers’ intention to purchase the autonomous product and ameliorates their evaluation of the outcome obtained with the help of the autonomous product. Still, these results only hold when consumers have no prior experience with the autonomous product. Jörling et al.’s findings offer insightful implications for researcher and marketers. First, they underline the meaning of product design for the perception and adoption of autonomous products which is in line with recent discussions of Raff et al. (2020). Second, by identifying anthropomorphism as a decisive component in the design of autonomous products especially for consumers with no prior product experience, they shed light on the psychological mechanisms underlying consumers perceptions of autonomous products.

The second article, “Mobile location-based services’ value-in-use in inner cities: Do a customer’s shopping patterns, prior user experience, and sales promotions matter?” by Stephanie Schwipper, Severine Peche, and Gertrud Schmitz, investigates value creation by means of smart services, here in the guise of mobile location-based services (LBS) connecting local customers, inner-city retailers and inner-city stakeholders via a local multi-sided digital community platform. On the basis of field data derived from actual users of a mobile LBS app in inner cities, the authors applied a mixed-method approach combining partial least squares structural equation modeling and fuzzy-set qualitative comparative analysis. They reveal that both utilitarian (e.g., monetary benefits) and hedonic (e.g., fun benefits) value-in-use components contribute to high value-in-use evaluations of mobile LBS usage. The meaning of some of the identified value-in-use components vary (i) over time due to an increase in user experience and (ii) at least to some extent for customers’ shopping patterns. Managers of mobile LBS can benefit from Schwipper et al.’s findings by acknowledging the importance of hedonic components such as fun benefits for customers’ value creation. However, utilitarian aspects of the mobile LBS are shown to be of equal importance: the possibility for customization of the app design and functionalities, for example, may contribute to user competence and therefore decrease the likelihood of customers’ perceptions of irritation (i.e., negative value-in-use component), when initially using the app. Thus, managers of mobile LBS are well advised to consider both hedonic and utilitarian components simultaneously.

The third article, “Creating value from energy data: A practitioner’s perspective on data-driven smart energy business models” by Friedrich Chasin, Ute Paukstadt, Patrick Ullmeyer, and Jörg Becker, explores the potential, the components, and the challenges of innovative business models for the end consumer market that use data created from smart energy products. Based on a qualitative study containing nine explorative semi-structured interviews with experts in various positions at companies in the energy domain, the authors offer a vision for consumer-oriented data-driven smart energy business models, which is constructed around five central categories: customer value, underlying infrastructure to deliver the value, competitive strategy, customer interface, and revenue model. For each category, Chasin et al. present several topics derived from the interviews and supported by academic research, which provide detailed information on possible specifications within the categories (e.g., different value propositions for the category “customer value”). On the basis of their findings, the authors also offer guiding implications for the design of data-driven smart energy business models, for example, by assessing the future potential of the identified value propositions. This discussion can serve as a meaningful starting point for energy companies that are about to create innovative, data-driven business models for the end consumer market.

The fourth article, “It takes more than two to tango: Identifying roles and patterns in multi-actor smart service innovation” by Jürgen Anke, Jens Pöppelbuß, and Rainer Alt, is concerned with smart service systems for which a network of actors, who are specialized in systems integration, user interface design, cloud computing, platform business, etc., is required in order to put them in place; these actors may assume several roles in the process simultaneously and may also actively change roles over time. Based on insights from an interview study, the authors describe (i) 17 roles involved in smart service systems engineering (SSSE) projects and (ii) actor-role constellations that reflect four patterns of smart service innovation. Practitioners can benefit from findings of Anke et al. in that they may use the provided lexicon of roles for both operational tasks, when planning an SSSE project and deciding whether these roles can be sourced internally or hired externally, and for strategic decisions, when selecting skills or knowledge, they want to build up within their firm.

To sum up, the four articles of the Special Section consider different crucial aspects of the management of smart products and services. While Jörling et al. focus on the meaning of the design of hardware components of smart products for the consumer perception, Schwipper et al. scrutinize utilitarian and hedonic value-in-use components to gain a better understanding of how value is created with smart services. The idea of value creation with the help of smart products and services, in turn, builds the foundation for consumer-oriented data-driven smart energy business models in the article of Chasin et al. Finally, Anke et al. adopt an ecosystems perspective to offer a better understanding of collaborating actors and their roles when working on smart service innovations. With these four perspectives, this Special Section provides insights on concepts in the domain of smart technologies as recently differentiated by Raff et al. (2020).

Overall, we feel that the strand of research dealing with managerial implications of smart products and related services is rapidly expanding. Corresponding topics concern conceptual aspects such as finding a common language when discussing smart products (e.g., Raff et al. 2020), customer relationship issues such as the meaning of trust for the adoption of and relationship building with smart products and services (e.g., Foehr and Germelmann 2020; Michler et al. 2020), issues of market introduction of new smart products (e.g., Decker and Stummer 2017), issues related to the development of innovative business models based on smart services (e.g., Dreyer et al. 2019; Wünderlich et al. 2015), organizational issues such as the establishment of digital innovation labs (e.g., Kaiser and Stummer 2020), (un)intended effects arising from the cooperation with startups that were set up in an attempt to better master the digital transformation (e.g., Schleef et al. 2020), or the meaning of smart technology infusion for the organizational frontline (e.g., De Keyser et al. 2019; Marinova et al. 2017). This list only provides a few examples of future research topics and does not claim to be complete. In light of the swift momentum of the field, there certainly will be another CHIMSPAS event in 2021 or 2022—depending on the COVID-19 situation. We would like to seize this opportunity to invite colleagues interested in the further development of research in the field of smart products and services to participate in and contribute to this conference.

## References

[CR1] Dawid H, Decker R, Hermann T, Jahnke H, Klat W, König R, Stummer C (2017). Management science in the era of smart consumer products: challenges and research perspectives. Central European Journal of Operations Research.

[CR2] Decker R, Stummer C (2017). Marketing management for consumer products in the era of the Internet of things. Advances in Internet of Things.

[CR4] Dreyer S, Olivotti D, Lebek B, Breitner MH (2019). Focusing the customer through smart services: a literature review. Electronic Markets.

[CR5] Fiske ST, Linville PW (1980). What does the schema concept buy us?. Personality and Social Psychology Bulletin.

[CR6] Foehr J, Germelmann CC (2020). Alexa, can I trust you? Exploring consumer paths to trust in smart voice-interaction technologies. Journal of the Association for Consumer Research.

[CR7] Kaiser I, Stummer C (2020). How the traditional industrial manufacturer Miele established a new smart home division. Research-Technology Management.

[CR3] De Keyser A, Köcher S, Alkire L, Verbeeck C, Kandampully J (2019). Frontline service technology infusion: conceptual archetypes and future research directions. Journal of Service Management.

[CR8] Mani Z, Chouk I (2018). Consumer resistance to innovation in services: challenges and barriers in the Internet of things era. Journal of Product Innovation Management.

[CR9] Marinova D, de Ruyter K, Huang M-H, Meuter ML, Challagalla G (2017). Getting smart: learning from technology-empowered frontline interactions. Journal of Service Research.

[CR10] Michler O, Decker R, Stummer C (2020). To trust or not to trust smart consumer products: a literature review of trust-building factors. Management Review Quarterly.

[CR11] Ng ICL, Walkenshaw SYL (2017). The Internet-of-things: review and research directions. International Journal of Research in Marketing.

[CR12] Panetta, Kasey. 2018. Gartner top 10 strategic technology trends for 2019. Published online October 15, 2018. https://www.gartner.com/smarterwithgartner/gartner-top-10-strategic-technology-trends-for-2019/. Accessed 28 Sept 2020.

[CR13] Porter MW, Heppelmann JE (2014). How smart, connected products are transforming competition. Harvard Business Review.

[CR14] Porter MW, Heppelmann JE (2015). How smart, connected products are transforming companies. Harvard Business Review.

[CR15] Raff, Stefan, Daniel Wentzel, and Nikolaus Obwegeser. 2020. Smart products: conceptual review, synthesis, and research directions. *Journal of Product Innovation Management*. 10.1111/jpim.12544

[CR16] Schleef M, Bilstein N, Stummer C (2020). “Shh! … I got help to become smart”: should incumbent firms disclose their cooperation with a startup?. Proceedings of the Forty-First International Conference on Information Systems (ICIS).

[CR17] Schweitzer F, van den Hende EA (2016). To be or not to be in thrall to the march of smart products. Psychology & Marketing.

[CR18] Souka M, Böger D, Decker R, Stummer C, Wiemann A (2020). Is more automation always better? An empirical study of customers’ willingness to use autonomous vehicle functions. International Journal of Automotive Technology and Management.

[CR19] Wünderlich NV, Heinonen K, Ostrom AL, Patrizio L, Sousa R, Voss C, Lemmink JGAM (2015). “Futurizing” smart service: implications for service researchers and managers. Journal of Services Marketing.

